# Safety and efficacy of tyrosine kinase inhibitors for the treatment of multiple sclerosis: A systematic review and meta-analysis from randomized controlled trials

**DOI:** 10.3389/fneur.2022.933123

**Published:** 2022-09-26

**Authors:** Zeya Yan, Feng Gu, Zilan Wang, Jiahao Meng, Xinyu Tao, Qiling Dai, Wei Wang, Meirong Liu, Zhong Wang

**Affiliations:** ^1^Department of Neurosurgery & Brain and Nerve Research Laboratory, The First Affiliated Hospital of Soochow University, Suzhou, China; ^2^Department of Neurology, The First Affiliated Hospital of Soochow University, Suzhou, China

**Keywords:** multiple sclerosis, gadolinium-enhancing lesions on MRI, meta-analysis, randomized controlled trials, tyrosine kinase inhibitor

## Abstract

**Background:**

Multiple sclerosis (MS), an autoimmune disease, is characterized by inflammatory demyelinating lesions in the white matter of the central nervous system. Drugs targeting tyrosine kinase, a critical component of immune cell receptor signaling, have been developed to treat MS. However, the exact efficacy and safety of tyrosine kinase inhibitors (TKIs) are still controversial, and comprehensive analysis with a high level of evidence is needed.

**Methods:**

Medline, Embase, Cochrane Library, and Clinicaltrials.gov for randomized controlled trials (RCTs) evaluating TKIs versus placebo for MS were searched up to April 1st, 2022. The risk ratio (RR) and mean difference (MD) or standard mean difference (SMD) were analyzed using dichotomous outcomes and continuous outcomes, respectively, with a random effect model.

**Results:**

A total of 1,043 patients derived from four clinical trials were included to investigate the efficacy and safety of TKI therapy for MS. According to our analysis, TKIs decreased the cumulative number of gadolinium-enhancing lesions on T1-weighted MRI with the application of high dose (SMD = −0.61, 95% CI: −0.93 to −0.30, *P* = 0.0001). Meanwhile, TKIs prevented the expanded disability status scale (EDSS) from rising (MD = −0.10, 95% CI: −0.19 to −0.00, *P* = 0.046). In terms of MS relapse, TKIs have not revealed an obvious statistical difference compared with placebo (RR = 0.96, 95% CI: 0.55–1.65, *P* = 0.8755). However, more adverse events seem to occur in the TKIs group, both for adverse events (RR = 1.12, 95% CI: 1.05–1.19, *P* = 0.0009) and serious adverse events (RR = 1.91, 95% CI: 1.30–2.81, *P* = 0.001).

**Conclusion:**

Tyrosine kinase inhibitors have shown promise in treating MS. Generally, TKIs that attain the effective dose demonstrate definite efficacy and have tolerable side effects. More clinical trials and validation are needed, and we anticipate that TKIs will be a viable alternative for MS patients.

## Introduction

Multiple sclerosis (MS) is one of the most common chronic neurological inflammatory diseases (1). With an incidence of 50–300 per 100,000 people, MS affects more than 2 million people globally, causing serious non-traumatic neurological disability that depends on the location of MS lesions (2). The symptoms usually appear in the early adult years of patients' lives, posing a significant risk to their quality and length of life (3). Magnetic resonance imaging is considered a reliable diagnostic for the diagnosis of MS and has been applied to track the progression of this disease (4, 5). To define patient groups, MS can be classified into two phenotypes: relapsing-remitting multiple sclerosis (RRMS) and progressive multiple sclerosis (PMS) (6). Moreover, a provisional disease course (relapsing-remitting, primary progressive, or secondary progressive) needs to be specified at the diagnosis time (7). Although therapies like glucocorticoid, plasma exchange, and others have been demonstrated to be effective for treating MS at the acute stage, they come with their own set of complications and limits as the disease progresses (8, 9). Therefore, MS is still incurable, and no therapy has been found to completely alleviate or prevent the progressive neurological disability (1). It is believed that new, effective treatments will be produced.

The pathogenesis of MS remains mysterious. Both the innate and adaptive immune systems play a momentous role in the complex pathogenesis process of MS. Tyrosine kinases have been implicated in the signaling of many immune cells and have been focused on new therapies for MS (10). B lymphocytes, in particular, have a role in the immunopathological aspects of MS. Treatments that target B cell depletion, such as CD20 antibodies, have already been shown to be effective in MS (11). Bruton's tyrosine kinase (BTK) is a member of the Tec family of kinases. As a pivotal component of B cell receptor signaling, BTK has a significant function in the processes of B cell maturation, activation, cell proliferation, and survival (12, 13). Mast cells and dendritic cells have also been proven to participate actively in the pathogenesis of MS (14, 15). The c-kit receptor tyrosine kinase activated stem cell factor (SCF), which is expressed on the mast cell surface, causes inappropriate proliferation, differentiation, and accumulation of mast cells (16). Based on this, inhibition of BTK and c-kit receptor tyrosine kinases is supposed to lessen the acute inflammation in the central nervous system caused by MS (10, 17).

So far, 13 tyrosine kinase inhibitors (TKIs) have been created and evaluated for the treatment of various diseases in at least phase 2 clinical trials, according to our knowledge, including autoimmune diseases such as rheumatoid arthritis or systemic lupus erythematosus, with promising results (18, 19). Recently, the application of TKIs, including Masitinib, Tolebrutinib, and Evobrutinib, in treating MS has caught researchers' attention (13, 20, 21). There has, however, been no systematic review and meta-analysis investigating the overall efficacy and safety of TKIs in treating MS.

Therefore, we conducted this meta-analysis of randomized controlled trials (RCTs) to investigate the efficacy and safety of various TKIs for treating MS. Moreover, we also focused on the impact of different TKI dosages on MS treatment.

## Methods

The procedure of our meta-analysis was conducted according to the Preferred Reporting Items for Systematic Reviews and Meta-Analyses (PRISMA) guidelines (22). This meta-analysis has not been registered.

### Search strategy

A systematic literature search was conducted by two authors independently up to April 1st, 2022. We searched databases including MEDLINE, Embase, and the Cochrane Library. We also searched Clinicaltrials.gov for more registered trials. In addition, we manually screened the reference lists of relevant articles to make sure all relevant studies were searched and included by us. A detailed search strategy is available in Supplemental materials.

### Eligible criteria

Inclusion criteria are as follows: (1) population: enrolled participants diagnosed with multiple sclerosis and all participants were over 18 years; (2) intervention: at least one type of TKIs was applied for the treatment of multiple sclerosis; (3) comparison: we did the comparison between different doses of TKIs group with the placebo group; (4) outcomes: objective indicators to measure the progression of multiple sclerosis. Exclusion criteria are as follows: (1) Essential data not available; (2) Studies in the form of comments, conferences, and abstracts or any have not been published in full text.

### Data extraction

The data extraction procedure was conducted by two authors independently, and a senior author reached a consensus on any disagreement that arose. For further analysis, we carefully collected the basic characteristics of studies, including author, publication year, publication, regions, centers, type of TKIs, treatment groups, total number of participants, study period, and outcome events. According to the treatment regime, patient data (gender, age, type of phenotype, time since MS onset, T2 hyperintense lesion volume, and EDSS score) were also collected. Inclusion, exclusion criteria, outcome assessments, and conclusions of the included studies were also collected.

### Outcomes

Efficacy endpoints are as follows: (1) primary outcomes: the change in the cumulative number of gadolinium-enhancing lesions on T1-weighted magnetic resonance imaging (MRI) (7). (2) Secondary outcomes: (1) the change in the expanded disability status scale (EDSS) score (23) and (2) the relapse rate.

Safety endpoints are included: adverse events (AEs) and serious adverse events (SAEs) reported by studies.

### Risk of bias

Two authors independently and carefully assessed a senior author and resolved the risk of bias of studies included and any disagreement. We followed the standard of the Cochrane Collaboration to assess the risk of bias for the studies, which included six aspects of bias: selection bias, performance bias, detection bias, attrition bias, reporting bias, and other biases. The procedure was conducted with Review Manager 5.3 software (the Cochrane Collaboration, Oxford, UK).

### Statistical analysis

We estimated mean differences (MDs) and standard mean differences (SMDs) for continuous outcomes and risk ratios (RRs) for dichotomous outcomes, both with 95% confidence intervals (CIs) (24, 25). Considering the existence of potential heterogeneity, we chose the random-effect model (24). We used the Cochran *Q*-test and *I*^2^ statistic to quantitatively assess the heterogeneity between studies, with *I*^2^ values exceeding 25, 50, and 75% representing low, middle, and high heterogeneity, respectively (26). A <0.05 *P*-value was considered significant for all analyses, and all tests were two-tailed. The procedure of statistical analysis was conducted with R 4.1.1 software.

## Results

### Search results

**Figure 1:**
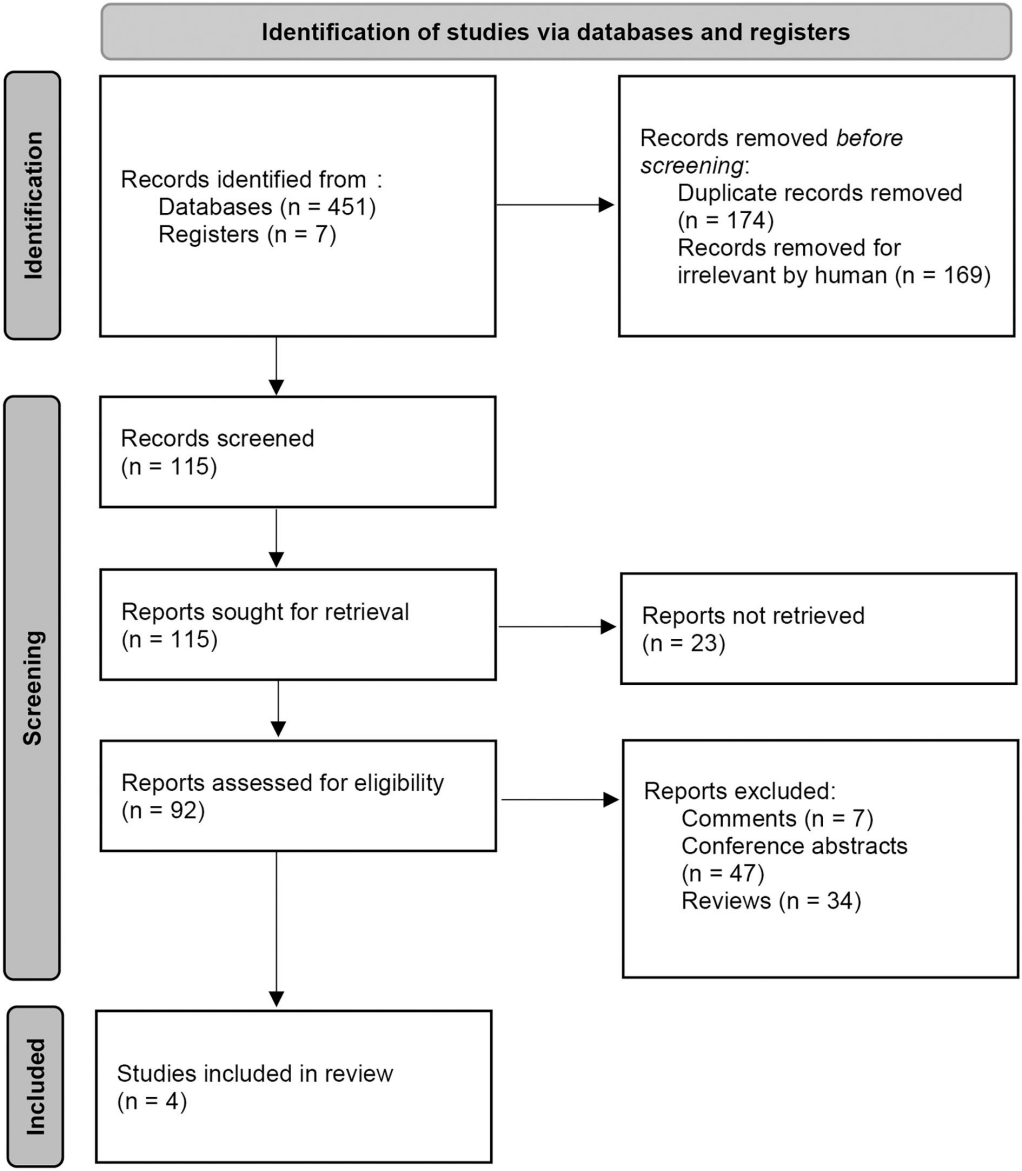
PRISMA flow gram of the study search, selection, and inclusion process.

A total of 458 records were identified from MEDLINE, Embase, the Cochrane Library, and Clinicaltrials.gov with a pre-formulated search strategy. One hundred seventy-four studies were duplicates, and another 169 studies were irrelevant and excluded. One hundred fifteen records were under review for the full article. Twenty-three records were not retrieved among them. Another 88 records were excluded, including seven comments, 47 conference abstracts, and 34 reviews. Finally, four RCTs containing a total of 1,043 patients were included in our meta-analysis. The detailed search procedure is presented in Figure 1.

### Study characteristics

All four studies were conducted as multi-center studies, with a total of 218 centers or hospitals included in our meta-analysis. Three different types of TKIs were applied to treat multiple sclerosis with different doses. The study period ranged from 16 weeks to 96 weeks. The detailed study characteristics are presented in Table 1. Moreover, the characteristics of different treatment regimes are presented in Table 2. Inclusion, exclusion criteria, outcome assessments, and conclusions of the included studies were available in the Supplementary Table S1.

**Table 1:** Characteristics of the included studies.

**Study**	**Regions**	**Centers**	**Publication**	**Type of Tyrosine** **kinase inhibitors**	**Treatment group,** **(number of participants)**	**Total number of** **participants (*n*)**	**Study period** **(weeks)**	**Outcome** **events**
Vermersch et al. (27) (NCT01433497)	Worldwide	116	Neurology Neuroimmunology and Neuroinfalmmation	Masitinib	Masitinib: 4.5 mg/kg/day (200); Placebo (101); Titrated masitinib: 6.0 mg/kg/day (203); tPlacebo (107)	611	96	a, b, e
Reich et al. (17) (NCT03889639)	Europe and North America	40	Lancet Neurology	Tolebrutinib	Tolebrutinib: 5 mg/day (33); 15 mg/day (32); 30 mg/day (33); 60 mg/day (32)	130	16	c, e
Montalban et al., 2019 (NCT02975349)	Europe	56	New England Journal of Medicine	Evobrutinib	Evobrutinib: 25 mg QD (50); 75 mg QD (51); 75 mg BID (53); Placebo (53); Dimethyl Fumarate (54)	267	48	a, b, c, e
Vermersch et al. (27) (Not applicable)	France	6	BMC Neurology	Masitinib	Masitinib: 3 mg/kg/day (12); 6 mg/kg/day (15); Placebo (8)	35	48	d, e

a, Least-squares mean change on the EDSS; b, Relapse rate; c, MRI T1 gadolinium-enhancing lesions; d, The average change in the MSFC score. e, Adverse events.

**Table 2:** Characteristics of the included tyrosine kinase inhibitors.

**Type of TKI**	**Treatment regime** **(*n*)**	**Gender** **(female %)**	**Age, year** **(mean ± SD)**	**Type of** **phenotype** **(*n*)**	**Time since MS onset,** **year (mean ± SD)**	**T2 hyperintense lesion** **volume, cm3 (mean ± SD)**	**EDSS score** **(mean ± SD)**
Masitinib	3.0 mg/kg/day (12) 6.0 mg/kg/day (15) 4.5 mg/kg/day (200) Titrated 6.0 mg/kg/day (203)	52^*^ 55.5 69.1	49 ± 9^*^ 49.8 ± 9.63 48.6 ± 10.10	PPMS (9) SPMS (15)^*^ PPMS (79) SPMS (120) PPMS (81) SPMS (122)	9.5 ± 7.3^*^ 14.0 ± 9.14 14.2 ± 9.96	N/A	4.9 ± 1.2^*^ 5.2 ± 1.07 5.5 ± 0.95
Tolebrutinib	5 mg/day (33); 15 mg/day (32); 30 mg/day (33); 60 mg/day (32)	76 66 64 75	36 ± 10 36 ± 9 39 ± 10 37 ± 9	RRMS (33) RRMS (32) RRMS (32) RRMS (31)	7.7 ± 7.8 8.0 ± 7.6 8.1 ± 7.8 7.3 ± 6.7	12.14 ± 9.86 11.74 ± 9.53 14.72 ± 13.80 12.41 ± 11.78	2.5 ± 1.0 2.2 ± 1.0 2.6 ± 1.3 2.7 ± 1.2
Evobrutinib	25 mg QD (50); 75mg QD (51); 75 mg BID (53)	64 69 68	42.4 ± 9.4 42.9 ± 10.1 42.2 ± 11.5	RRMS (42) SPMS (8) RRMS (43) SPMS (8) RRMS (47) SPMS (6)	10.85 ± 5.84 11.95 ± 5.38 14.95 ± 8.65	13.79 ± 11.67 14.03 ± 12.23 19.02 ± 13.54	3.3 ± 1.5 3.5 ± 1.4 3.4 ± 1.6

* Total of 3.0 mg/kg/day and 6.0 mg/kg/day.

TKI, tyrosine kinase inhibitors; MS, multiple sclerosis; EDSS, expanded disability status scale; RRMS, relapsing-remitting multiple sclerosis; SPMS, secondary progressive multiple sclerosis; PPMS, primary progressive multiple sclerosis.

### Primary efficacy outcome of TKIs in MS and subgroup analysis

**Figure 2:**
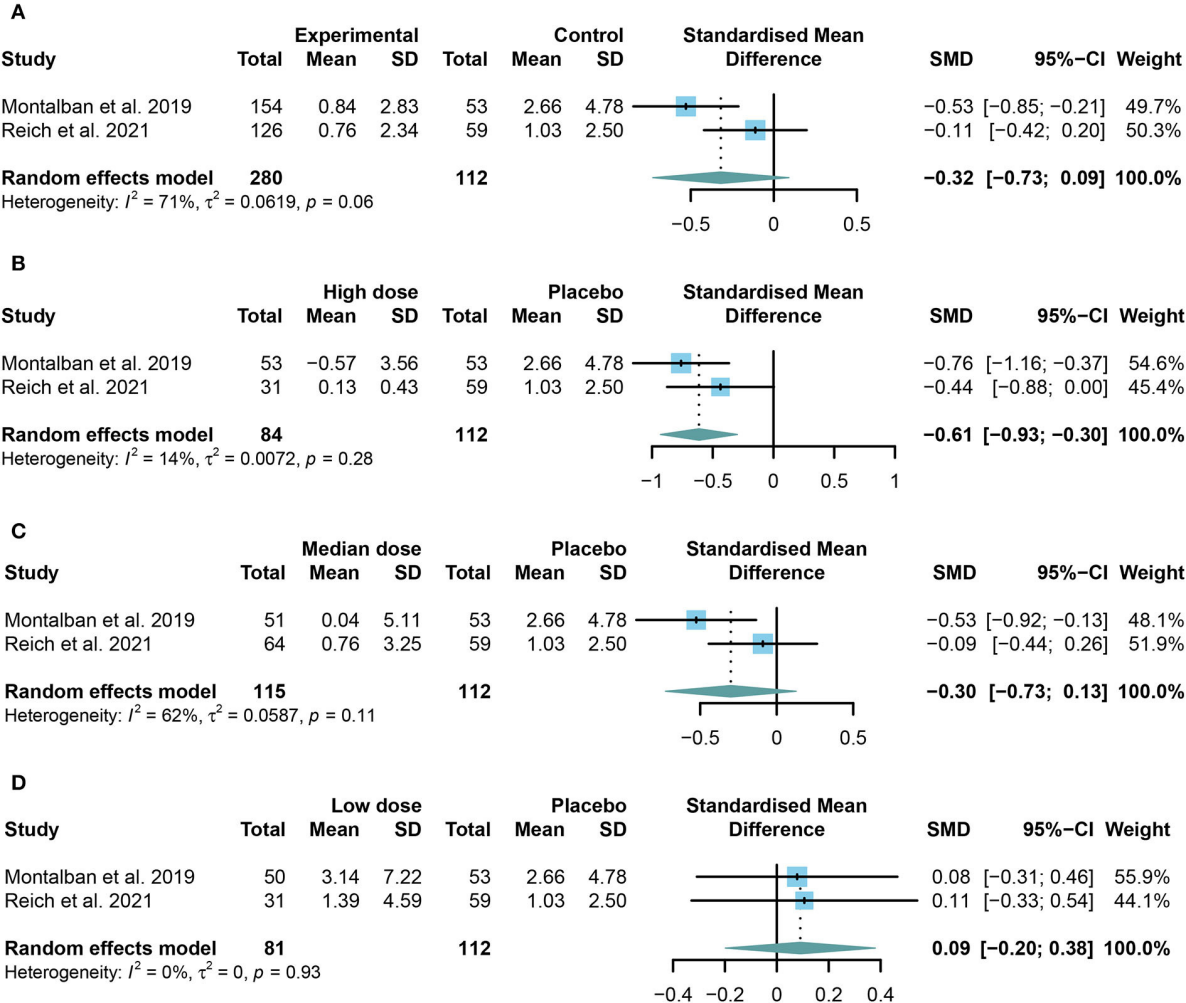
The pooled standard mean differences (SMDs) of the change in the cumulative number of gadolinium-enhancing lesions on T1-weighted MRI in different doses of TKIs compared with the placebo group; the diamond indicates the estimated summary SMDs with a 95% confidence interval (CI): **(A)** summary of different doses of TKIs compared with the placebo group; **(B)** high dose of TKIs compared with the placebo group; **(C)** median dose of TKIs compared with the placebo group; **(D)** low dose of TKIs compared with the placebo group.

The change in the cumulative number of gadolinium-enhancing lesions on T1-weighted MRI was designed as the primary efficacy outcome. However, no obvious difference was found in the change in the cumulative number of gadolinium-enhancing lesions on T1-weighted MRI between the TKIs group and the placebo group (SMD = −0.32, 95% CI: −0.73 to 0.09, *P* = 0.13; Figure 2A).

To further investigate the effect of TKIs, we compared the outcomes of different doses. Restricted to the integrality of data, we conducted a further study only on the change in the cumulative number of gadolinium-enhancing lesions on T1-weighted MRI. The high or low dose group is the highest or lowest group in each study. The median group refers to the groups between the highest and the lowest group. The results showed that patients in the high dose group performed significantly better than those in the placebo group (SMD = −0.61, 95% CI: −0.93 to −0.30, *P* = 0.0001) (Figure 2B). However, the low dose group (SMD = 0.09, 95% CI: −0.20 to 0.38, *P* = 0.54) or the median dose group (SMD = −0.30, 95% CI: −0.73 to 0.13, *P* = 0.17) had no significant effect on preventing the development of enhanced lesions compared with the placebo group (Figures 2C,D).

### Secondary efficacy outcome of TKIs in MS

**Figure 3:**
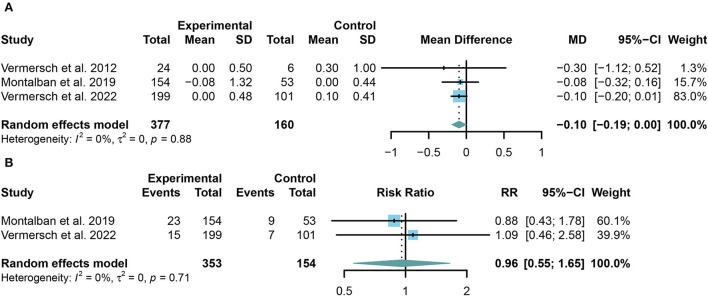
**(A)** The pooled mean differences (MDs) of the change in the expanded disability status scale (EDSS) score compared with the placebo group; **(B)** the pooled risk ratios (RRs) of the relapse rate of multiple sclerosis compared with the placebo group, the diamond indicates the estimated summary MD or RR with 95% confidence interval (CI).

TKIs showed significant efficacy in preventing the increase of EDSS (MD = −0.10, 95% CI: −0.19 to 0.00, *P* = 0.046). However, no significant change was found in the multiple sclerosis relapse (RR = 0.96, 95% CI: 0.55 to 1.65, *P* = 0.8755) between the summary TKIs group and the placebo group. The outcomes were presented in the form of a forest plot in Figures 3A,B.

### Safety outcomes of TKIs in MS

**Figure 4:**
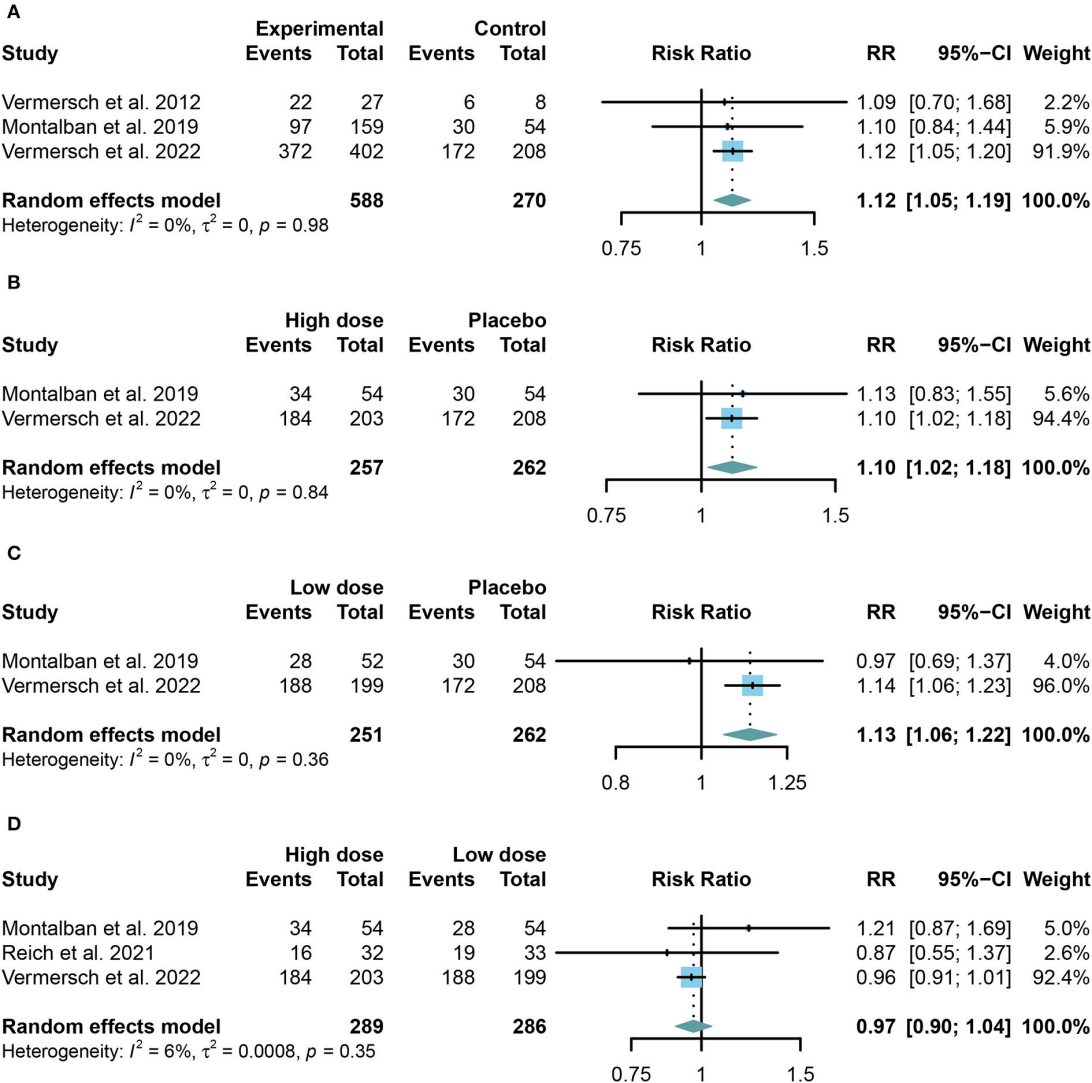
The pooled risk ratios (RRs) of patients with adverse events in different treatment doses compared with the placebo group; the diamond indicates the estimated summary RRs with a 95% confidence interval (CI). **(A)** Summary of different doses of TKIs compared with the placebo group; **(B)** high dose of TKIs compared with the placebo group; **(C)** low dose of TKIs compared with the placebo group; and **(D)** high dose of TKIs compared with low dose group.

**Figure 5:**
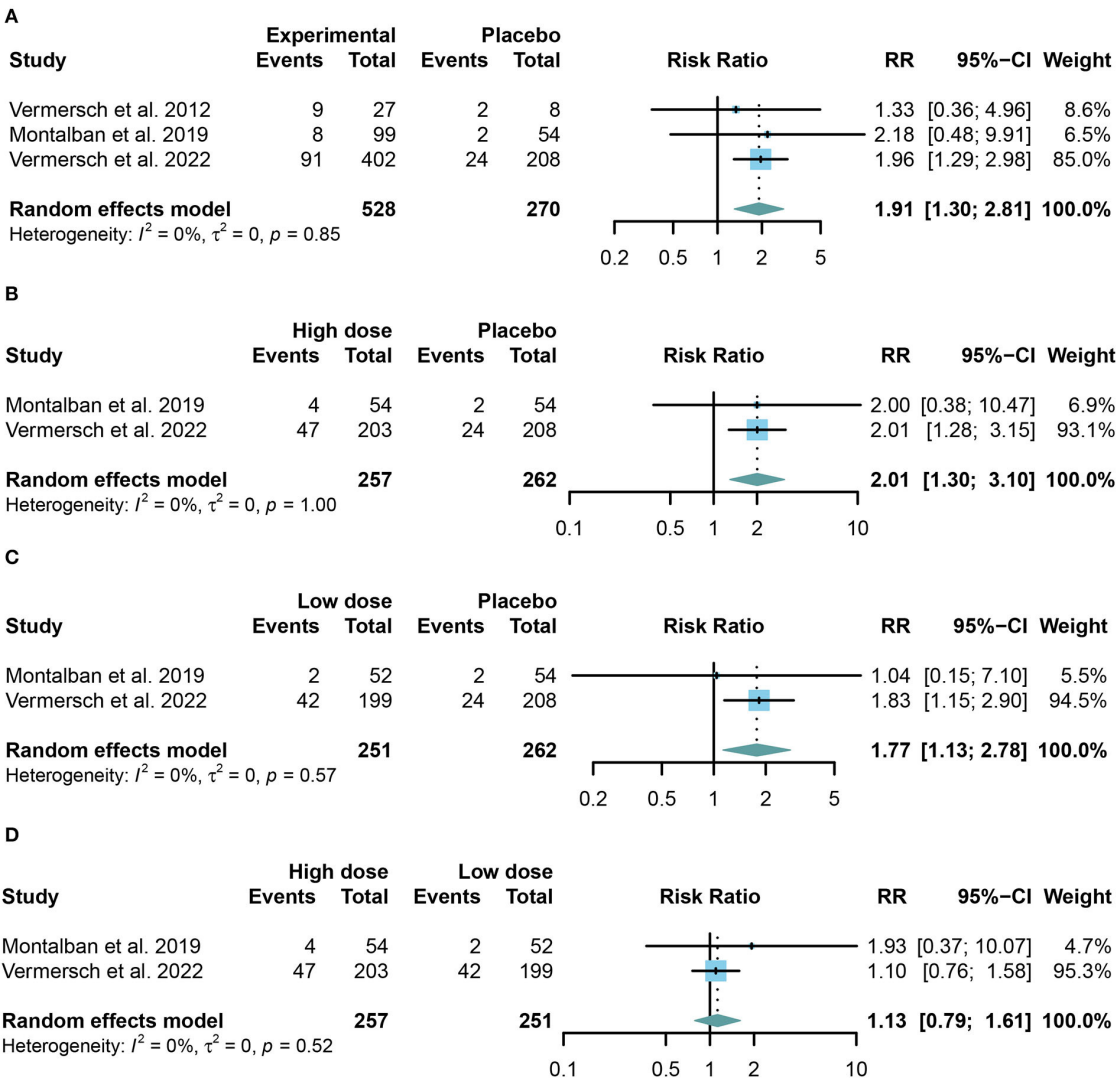
The pooled risk ratios (RRs) of patients with serious adverse events in different treatment doses compared with the placebo group; the diamond indicates the estimated summary RRs with a 95% confidence interval (CI). **(A)** Summary of different doses of TKIs compared with the placebo group; **(B)** high dose of TKIs compared with the placebo group; **(C)** low dose of TKIs compared with the placebo group; and **(D)** high dose of TKIs compared with the low dose group.

We combined the data of reported adverse events into a summary from all trials using random-effects models. Common adverse events in the trials include headache, upper respiratory tract infection, nasopharyngitis, and respiratory tract infection. Taking in TKIs has a higher risk of both AE and SAE than the placebo group (For AE: RR = 1.12, 95% CI: 1.05–1.19, *P* = 0.0009, Figure 4A; for SAE: RR = 1.91, 95% CI: 1.30–2.81, *P* = 0.001, Figure 5A). Similar results were found in the separate dose group compared with the placebo group (Figures 4B,C, 5B,C). However, we did not find any significant change between the high dose group and the low dose group in the incidence of both AE and SAE (For AE: RR = 0.97, 95% CI: 0.90–1.04, *P* = 0.39, Figure 4D; for SAE: RR = 1.13, 95% CI: 0.79–1.61, *P* = 0.52, Figure 5D).

### Outcomes of subgroup analysis

After analyzing the available data, we also conducted a subgroup analysis to further explore EDSS, AE, and SAE. As for the three TKIs, respectively, there seemed to be no obvious difference (*P* > 0.05). Results of this part can be found in the Supplementary Figures S1–S3.

### Risk of bias

**Figure 6:**
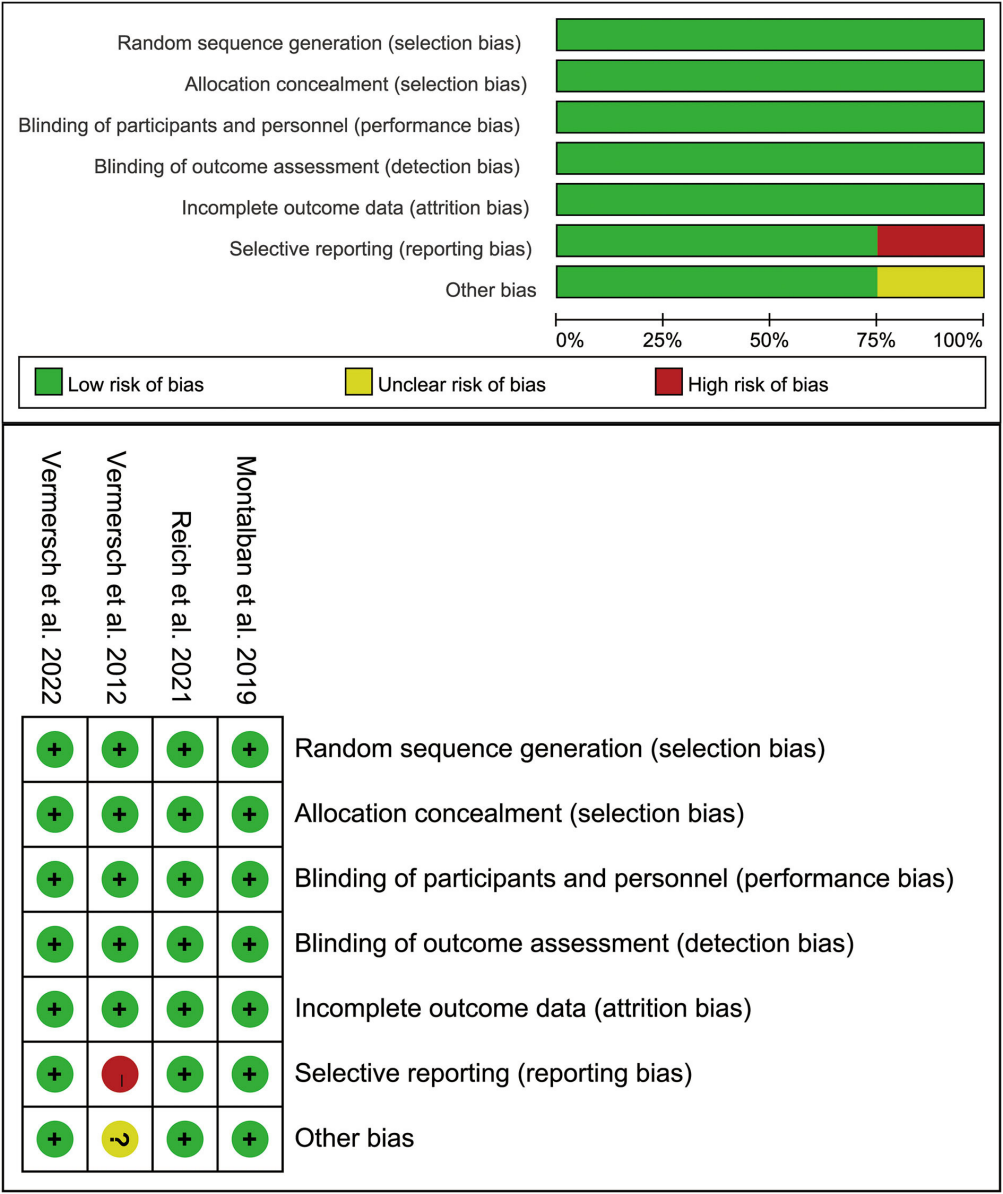
Summary table for potential bias analysis for the included study.

Detailed data on the risk bias are presented in Figure 6. Three of the four clinical trials showed low risks of bias. However, the study conducted by Vermersch et al. in 2012 showed a high risk of reporting bias for the incomplete report of their potential limitations, which failed to included results of a key outcomes. Moreover, though the study was conducted in six centers, the small sample still raised our concern about an unclear bias.

## Discussion

In our systematic review and meta-analysis, we have pooled 1,043 patients from four clinical trials to investigate the efficacy and safety of TKIs for MS therapy. According to our analysis, we discovered that TKIs have various advantages and risks when compared with placebo. Specifically, TKIs decrease the cumulative number of gadolinium-enhancing lesions on T1-weighted MRI with the application of a high dose. Meanwhile, TKIs prevent EDSS from rising. In terms of MS relapse, TKIs have not revealed a noticeable statistical difference compared with placebo. However, notably, more adverse events seem to occur in the TKIs group.

Bruton's tyrosine kinase causes B-cell and myeloid cell activation and pro-inflammatory polarization by transmitting signals through multiple receptors (19). BTK inhibitors were believed to lessen acute inflammatory reactions in MS pathology by blocking this pathway. However, based on our analysis of gadolinium-enhancing lesions on T1-weighted MRI and the objective reflection of focal inflammatory lesions, there exists no explicit difference in decreasing tendencies between the TKIs group and the placebo group contradicts the findings of evobrutinib and tolebrutinib. As a result, we conducted a subgroup analysis using a variety of dosages to investigate the difference between high and low doses. The results indicate that a high dose has an effect over a low dose. The explanation may point to the dose-dependent character of TKI occupancy, such as single evobrutinib doses of greater than or equal to 100 mg with more than 50% of TKI occupancy (28). Similarly, tolebrutinib at 60 mg/day has been confirmed to be effective in reducing gadolinium-enhancing lesions (4). To put it another way, we obtained optimum efficacy at a specific blood concentration. As for the novel selective tyrosine kinase inhibitor, masitinib has no evaluation of gadolinium-enhancing lesions on T1-weighted MRI in existing research. We look forward to more studies exploring the difference between 4.5 mg/kg and 6.0 mg/kg daily for the application of masitinib.

Expanded disability status scale is one of the first standardized tools and is also the most frequently used scale for the evaluation of MS disability currently (29). Based on the analysis results of masitinib and evobrutinib, TKIs reveal the outstanding performance of EDSS, while there is no obvious difference between the three TKIs. Because TKIs reduce the acute immune response, it appears that they could benefit the long-term neurological function of MS patients. In terms of MS recurrence rate, analyzable data from TKIs above demonstrates no statistically significant difference compared to placebo. Because of its pharmacokinetic features, evobrutinib may not reduce the relapse rate. Evobrutinib's superiority for MS remission may be limited by its short half-life of 2 h and 85.3% excretion in the first 72 h after administration (28). Masitinib, which has been proven to maintain remission of mastocytosis for over two years, still has no specific explanation. Further studies are needed to better understand the details of masitinib's observed therapeutic benefit, just like the remodeling function of the neuronal microenvironment (30, 31).

From a security standpoint, TKIs appear more likely than placebos to have negative effects. In our results for TKIs, both AE and SAE have a greater incidence than a place. However, here is the good news: most AEs are rash, nausea, dizziness, and so on, which are no obvious difference from previous therapies, suggesting an acceptable safety. As for the SAE, the occurrence may be too small to indicate statistical significance, such as neutropenia, maculopapular rash, and elevations in ALT, AST, and lipase. We considered that safety margins for these TKIs have been improved by reforming selectivity, unique enzyme coverage characteristics, and pharmacokinetic profiles (32). It is worth noting that high dose and high dosing frequency seem more related to the occurrence of AEs. However, the analysis of AE for the high dose and low dose has not indicated any sense. Therefore, there may be some possible correlation between the dosing frequency and the occurrence of AE. More research directed at various dosing frequencies needs to be conducted for this.

Our systematic review and meta-analysis were performed to evaluate the new generation of TKIs holistically so that a more representative conclusion for the three TKIs included was obtained by superposition analysis of different species. Indeed, there still be some limitations. Firstly, the number of researchers and total sample size included is small, and the patient number in the latest study about masitinib is more than 50% of the total population (27). However, fortunately, it is enough for analysis to get some meaningful conclusions with low heterogeneity in most analyses. Moreover, outcomes reported were mostly enrolled in two of the three TKIs except for AE. Therefore, the conclusion we reached needs further verification for its universality. Last but not least, the study period included studies with a large period, which may increase the risk of bias.

## Conclusion

Collectively, TKIs included in our research, Masitinib, Tolebrutinib, and Evobrutinib, show promise in treating MS. TKIs that reach the effective dose demonstrate remarkable effectiveness, and adverse reactions are within tolerable limits. More clinical trials and validation need to be conducted for the mechanism and clinical efficacy of TKIs.

## Data availability statement

The original contributions presented in the study are included in the article/Supplementary material, further inquiries can be directed to the corresponding authors.

## Author contributions

ZW and ML were the major investigators. ZY and FG conceptualized the study and devised an analysis strategy. ZiW and JM evaluated the data and conducted the meta-analysis. ZY and XT contributed to the article's composition. QD and WW revised the manuscript and polished the language. All authors contributed to the article and approved the submitted version.

## Funding

This work was supported by the Suzhou Health Talents Training Project (GSWS2019002).

## Conflict of interest

The authors declare that the research was conducted in the absence of any commercial or financial relationships that could be construed as a potential conflict of interest.

## Publisher's note

All claims expressed in this article are solely those of the authors and do not necessarily represent those of their affiliated organizations, or those of the publisher, the editors and the reviewers. Any product that may be evaluated in this article, or claim that may be made by its manufacturer, is not guaranteed or endorsed by the publisher.
