# Supplementary material for: Safety and efficacy of tyrosine kinase inhibitors for the treatment of multiple sclerosis: A systematic review and meta-analysis from randomized controlled trials
Source: Front Neurol. 2022 Sep 26;13:933123. doi: 10.3389/fneur.2022.933123 (PMC9548566; doi:10.3389/fneur.2022.933123)
Supplement: Supplementary file 1 [file Data_Sheet_1.docx]

Supplementary Material

# Supplementary Data-Search Strategy

((tyrosine kinase inhibitor) OR (Bruton tyrosine kinase inhibitor) OR (BTK inhibitor) OR (evobrutinib) OR (Masitinib) OR (tolebrutinib) OR (BMS-986142) OR (Branebrutinib) OR (Acalabrutinib) OR (Elsubrutinib) OR (Evobrutinib) OR (Fenebrutinib) OR (Ibrutinib) OR (Poseltinib) OR (Remibrutinib) OR (Rilzabrutinib) OR (Spebrutinib) OR (Tirabrutinib) OR (Tolebrutinib)) AND (multiple sclerosis)

# Supplementary Tables

# Table S1 Inclusion, exclusion criteria, outcome assessments and conclusions of the included studies

| Trials | Vermersch et al. 2022  (NCT01433497) |
| --- | --- |
| *Inclusion criteria* | Key eligibility criteria were an age of 18–75 years, MS diagnosis (regardless of time from onset) according to the revised McDonald criteria of PPMS or nSPMS without relapse for at least 2 years prior to inclusion, and a baseline score on the EDSS of 2.0–6.0 inclusive (range, 0 to 10.0 in 0.5-point increments, with higher scores indicating greater disability). In addition, clinical evidence (medical record) of disability progression over the preceding 2 years (as measured by an increase in the EDSS score of at least 1.0 point) was required. The targeted population therefore comprised patients with progressive MS who are progressing but not clinically active (i.e., evidence of disability accumulation over time without relapses). |
| *Exclusion*  *criteria* | Key exclusion criteria were concurrent/recent use of immunomodulators, immunosuppressants, interferon beta-1, glatiramer acetate, corticoste roids, or any investigational drug with predefined wash-out periods to avoid potential confounding effects (see eMethods for further details, links.lww.com/NXI/A699). |
| *Efficacy outcomes* | 1. The primary end point was change from baseline on the EDSS   2. Other secondary end points included change from baseline on the MSFC raw scores averaged for all time points over 96 weeks, and its component measures of timed 25-foot walk test (T25FW, averaged time from 2 tests), 9-hole peg test (9-HPT, averaged time from 2 tests on each hand), and Paced Auditory Serial Addition Test–3 (PASAT-3). |
| *Safety outcomes* | The safety data set comprised all patients who received at least 1 dose of study medication. Patients were monitored for safety from date of informed consent until 28 days after discontinuing the study drug。 |
| *Conclusions* | In conclusion, masitinib at 4.5 mg/kg/d can benefit patients by slowing EDSS-based disability worsening; however, validation of these findings via a confirmatory phase 3 study will be necessary, in part because neuroimaging data were not collected during the current study and also due to an absence of signal on secondary end points. Overall, study AB07002 represents the first successful randomized, controlled, phase 3 trial in progressive MS of a tyrosine kinase inhibitor, targeting innate immune cells. |

| Trials | Reich et al. 2021  (NCT03889639) |
| --- | --- |
| *Inclusion criteria* | Eligibility criteria for participation in the trial included age 18–55 years with a diagnosis of relapsing multiple sclerosis (either relapsing-remitting or relapsing secondary progressive multiple sclerosis) according to the 2013 clinical course revisions, as specified in the 2017 McDonald diagnostic criteria (this is also consistent with the 2020 clarification of the 2013 clinical course descriptors). Participants also had to meet one or more of the following criteria: at least one relapse within the previous year, at least two relapses within the previous 2 years, or at least one active gadolinium-enhancing brain lesion in the 6 months before screening. |
| *Exclusion*  *criteria* | Exclusion criteria included a diagnosis of primary progressive multiple sclerosis, a diagnosis of secondary progressive multiple sclerosis without relapse, an Expanded Disability Status Scale (EDSS) score of more than 5.5 at  screening, and relapses occurring within 30 days of random allocation to treatment. |
| *Efficacy outcomes* | The primary efficacy endpoint was the number of new gadolinium-enhancing lesions detected on the scan done after 12 weeks of tolebrutinib treatment (at week 12 for cohort 1 and week 16 for cohort 2), relative to the scan done 4 weeks previously. Secondary endpoints were the number of new or enlarging T2 lesions detected on the same scan after 12 weeks of treatment, the total number of gadoliniumenhancing lesions on the scan at the end of 12 weeks of tolebrutinib treatment, |
| *Safety outcomes* | Adverse events, serious adverse events, and adverse events of special interest based on class effects: bleeding, cytopenias, infections, atrial arrhythmias, and liver enzyme elevations. |
| *Conclusions* | In summary, our study design with short placebo exposure established an effect of tolebrutinib on MRI measures related to new lesion formation and identified a dose to test in phase 3 trials. Effective treatment for acute inflammation, combined with the potential to directly modulate the immune response within the CNS—known to be a key driver of clinical progression in multiple sclerosis—provides scientific rationale to pursue phase 3 clinical trials in patients with both relapsing and progressive forms of multiple sclerosis. |

| Trials | Montalban et al. 2019  (NCT02975349) |
| --- | --- |
| *Inclusion criteria* | Patients were eligible for treatment if they were between the ages of 18 and 65 years, had relapsing–remitting multiple sclerosis or secondary progressive multiple sclerosis with superimposed relapses,17,18 and had a score of no more than 6 on the EDSS (which ranges from 0 [no disability] to 10 [death]); all the patients provided written informed consent. |
| *Exclusion*  *criteria* | Key exclusion criteria were progressive multiple sclerosis, either primary or secondary with no superimposed relapses; a disease duration of more than 15 years with an EDSS score of 2 or less; and exposure to DMF within 6 months before randomization. |
| *Efficacy outcomes* | The primary end point was the total (cumulative) number of gadolinium-enhancing lesions identified on T1-weighted MRI at weeks 12, 16, 20, and 24. Key secondary end points were the annualized relapse rate, based on qualified relapses; qualified relapse-free status; change from baseline in the EDSS score at week 24. |
| *Safety outcomes* | A qualified relapse was defined as new, worsening, or recurrent neurologic symptoms attributed to multiple sclerosis that lasted for at least 24 hours without fever, infection, or adverse reaction to a prescribed medication and that was preceded by a stable or improving neurologic status of at least 30 days. |
| *Conclusion* | In conclusion, in patients with relapsing multiple sclerosis, the inhibition of BTK with evobrutinib at a dose of 75 mg once daily, but not at doses of 75 mg twice daily or 25 mg once daily, reduced the total number of enhancing MRI lesions, as compared with placebo, at weeks 12 through 24. Treatment with evobrutinib at any dose had no effect on the annualized relapse rate or disability progression and was associated with elevations in liver amino transferase levels. |

| Trials | Vermersch et al. 2012  (Not applicable) |
| --- | --- |
| *Inclusion criteria* | Patients aged 18 to 60, suffering from PPMS or rfSPMS as diagnosed by the ‘McDonald criteria’and having an Expanded Disability Status Scale (EDSS) score between 2 to 6.5 with a progression > 1 within 2 years prior to inclusion, were eligible for this study. |
| *Exclusion*  *criteria* | The following conditions were exclusion criteria: patients having SPMS with relapse in the 2 years before inclusion; treatment with interferon, glatiramer, oral or systemic corticosteroids, adrenocorticotropic hormone, or an investigational agent within 4 weeks of inclusion; and inadequate organ function defined via blood test levels. |
| *Efficacy outcomes* | Evaluation of treatment effect was based upon change in clinical neurological functions. The primary endpoint was the average change in multiple sclerosis functional composite (MSFC) score relative to baseline, with clinical response defined as a >100% improvement (increase) from baseline. The MSFC score is a multidimensional, MS-specific outcome measure, comprising of a timed 25-foot walk (T25FW) test measuring leg function and ambulation, a nine hole peg test (9-HPT) measuring arm and hand function and a Paced Auditory Serial Addition Test 3 seconds (PASAT-3) measuring cognitive function. The MSFC was calculated as described in the National Multiple Sclerosis Society MSFC administration and scoring manual. Secondary endpoints included analysis of the MSFC subcategories (namely, T25FW, 9-HPT and PASAT-3), and the expanded disability status scale(EDSS). |
| *Safety outcomes* | Safety was assessed throughout the study via physical examinations, vital signs, clinical laboratory evaluations and monitoring of adverse events (AEs), with all AEs recorded regardless of causality. |
| *Conclusion* | Thus, masitinib’s anti-mast cell properties, and possible effect on dendritic cells, may be particularly well adapted to the treatment of PPMS. A reduction of mast cell activity via the inhibitory action of masitinib on c-Kit, Lyn and Fyn tyrosine kinase activity, impacting both inflammatory mediated and NO-mediated damage mechanisms, while inhibition of dendritic cell activity may disrupt the signaling pathways relevant to T helper cells. The findings of the current study, within limitations inherent to such an exploratory trial, suggest that oral masitinib was relatively well tolerated and can be of therapeutic potential in the treatment of MS, with positive responses observed in  some relevant measures of this condition. Moreover, this positive action was observed in patients with PPMS and rfSPMS, subpopulations for whom there are practically no currently available treatments. Taken together with positive results from the complementary EAE mouse model, this trial provides evidence that supports a larger placebocontrolled investigation. |

| **Adverse Events** | **Tyrosine Kinase Inhibitors** | | **Placebo** | | **Risk Ratio** | **95%CI** | **P-value** | **I^2^(%)** |
| --- | --- | --- | --- | --- | --- | --- | --- | --- |
|  | **n** | **total** | **n** | **total** |  |  |  |  |
| Diarrhea | 30 | 358 | 7 | 155 | 0.88 | [0.06,13.96] | 0.93 | 79 |
| Nausea | 24 | 358 | 4 | 155 | 2.63 | [0.99,7.04] | 0.05 | 0 |
| Peripheral oedema | 19 | 329 | 5 | 231 | 2.44 | [0.30,19.78] | 0.40 | 67 |
| Low lymphocyte count | 23 | 358 | 6 | 155 | 1.79 | [0.77,4.14] | 0.18 | 0 |
| Nasopharyngitis | 24 | 289 | 10 | 184 | 1.17 | [0.56,2.46] | 0.67 | 0 |
| Headache | 15 | 289 | 11 | 184 | 1.00 | [0.46,2.18] | 0.99 | 0 |
| Upper respiratory tract infection | 50 | 488 | 26 | 285 | 1.07 | [0.69,1.67] | 0.75 | 0 |

**Table S2. Detailed analysis of common adverse events.**

# Supplementary Figures

**
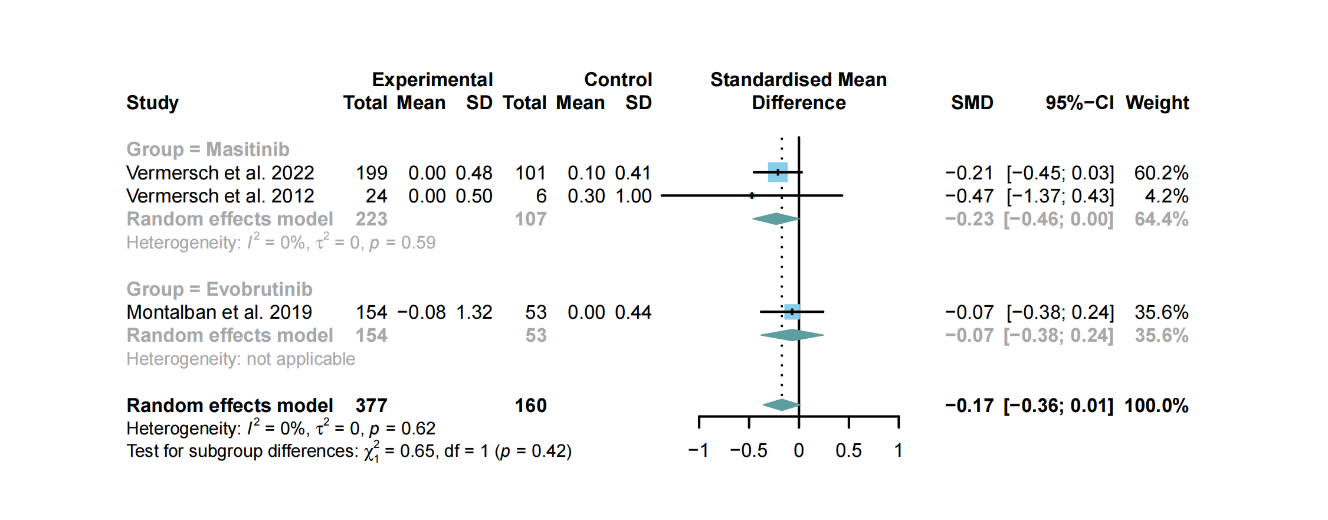
Figure S1 Subgroup analysis of Expanded Disability Status Scale**


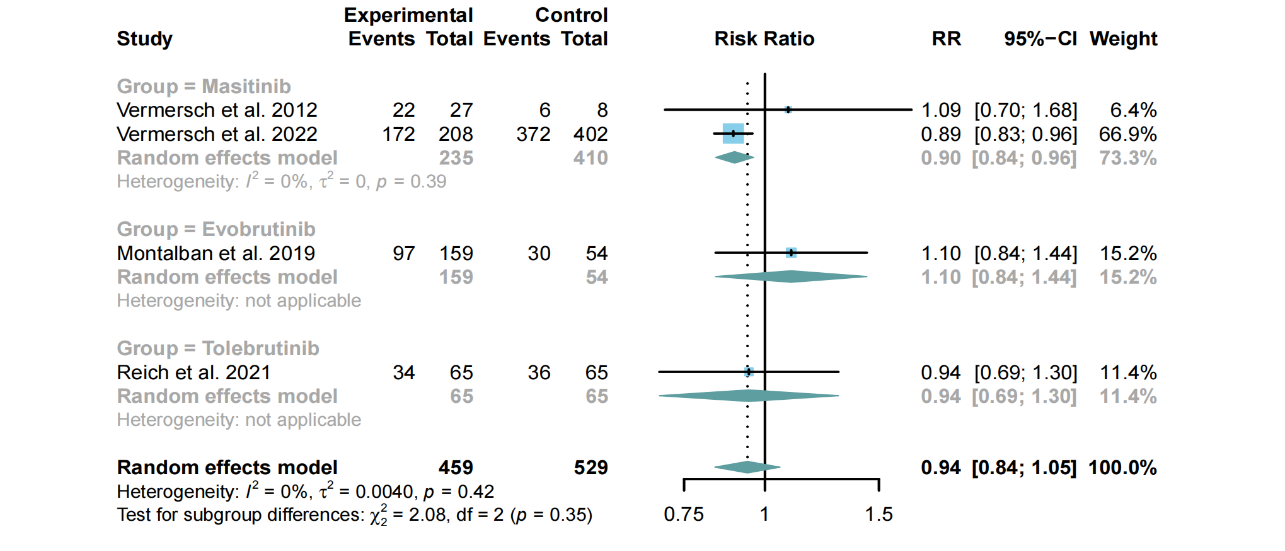
**Figure S2 Subgroup analysis of adverse events**


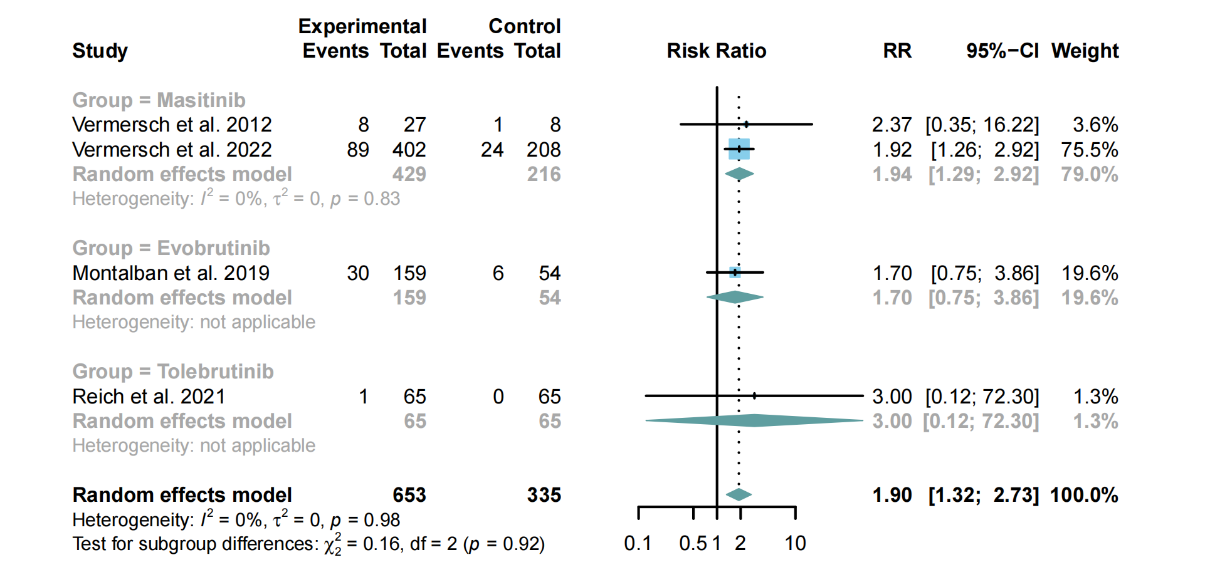
**Figure S3 Subgroup analysis of serious adverse events**
